# Associations of severe adverse perinatal outcomes among continuous birth weight percentiles on different birth weight charts: a secondary analysis of a cluster randomized trial

**DOI:** 10.1186/s12884-022-04680-5

**Published:** 2022-04-30

**Authors:** Hester D. Kamphof, Sanne J. Gordijn, Wessel Ganzevoort, Viki Verfaille, Pien M. Offerhaus, Arie Franx, Eva Pajkrt, Ank de Jonge, Jens Henrichs

**Affiliations:** 1 Department of Obstetrics and Gynecology, University Medical Center Groningen, University of Groningen, Groningen, The Netherlands; 2 Department of Obstetrics, Amsterdam UMC, University of Amsterdam, Amsterdam Reproduction and Development Research Institute, Amsterdam, Netherlands; 3 Dutch Professional Association of Sonographers (BEN), Woerden, the Netherlands; 4 AVM (Midwifery Education and Studies Maastricht, ZUYD University of Applied Sciences), Maastricht, the Netherlands; 5 Department of Obstetrics and Gynecology, Erasmus Medical Center, Rotterdam, Netherlands; 6 Department of Midwifery Science, Amsterdam University Medical Centers, Vrije Universiteit Amsterdam, AVAG/Amsterdam Public Health, Amsterdam, Netherlands

**Keywords:** Birth weight standard, Birth weight reference charts, Birth weight reference curve, Fetal growth restriction, FGR, IUGR, Growth restriction in the newborn, Small for gestational age, SGA

## Abstract

**Objective:**

To identify neonatal risk for severe adverse perinatal outcomes across birth weight centiles in two Dutch and one international birth weight chart.

**Background:**

Growth restricted newborns have not reached their intrinsic growth potential in utero and are at risk of perinatal morbidity and mortality. There is no golden standard for the confirmation of the diagnosis of fetal growth restriction after birth. Estimated fetal weight and birth weight below the 10^th^ percentile are generally used as proxy for growth restriction. The choice of birth weight chart influences the specific cut-off by which birth weight is defined as abnormal, thereby triggering clinical management. Ideally, this cut-off should discriminate appropriately between newborns at low and at high risk of severe adverse perinatal outcomes and consequently correctly inform clinical management.

**Methods:**

This is a secondary analysis of the IUGR Risk Selection (IRIS) study. Newborns (*n* = 12 953) of women with a low-risk status at the start of pregnancy and that received primary antenatal care in the Netherlands were included.

We examined the distribution of severe adverse perinatal outcomes across birth weight centiles for three birth weight charts (Visser, Hoftiezer and INTERGROWTH) by categorizing birth weight centile groups and comparing the prognostic performance for severe adverse perinatal outcomes.

Severe adverse perinatal outcomes were defined as a composite of one or more of the following: perinatal death, Apgar score < 4 at 5 min, impaired consciousness, asphyxia, seizures, assisted ventilation, septicemia, meningitis, bronchopulmonary dysplasia, intraventricular hemorrhage, periventricular leukomalacia, or necrotizing enterocolitis.

**Results:**

We found the highest rates of severe adverse perinatal outcomes among the smallest newborns (< 3^rd^ percentile) (6.2% for the Visser reference curve, 8.6% for the Hoftiezer chart and 12.0% for the INTERGROWTH chart). Discriminative abilities of the three birth weight charts across the entire range of birth weight centiles were poor with areas under the curve ranging from 0.57 to 0.61. Sensitivity rates of the various cut-offs were also low.

**Conclusions:**

The clinical utility of all three charts in identifying high risk of severe adverse perinatal outcomes is poor. There is no single cut-off that discriminates clearly between newborns at low or high risk.

**Trial Registration:**

Netherlands Trial Register NTR4367.

Registration date March 20^th^, 2014.

## Background

Fetal growth restriction (FGR) refers to a condition in which a fetus does not reach its intrinsic growth potential, usually due to placental insufficiency [1]. Unrecognized FGR increases the risk of stillbirth and severe complications [2, 3]. After birth, growth restricted newborns are at risk for perinatal mortality and morbidity and somatic and neurological complications [4–14].

In the absence of a gold standard for the diagnosis of growth restriction in the newborn, small-for-gestational-age (SGA) is used as a screening tool. However, SGA represents a statistical deviation of birth weight from a population-based reference, predefined as a cut-off that is generally the 10^th^ percentile [14]. No international consensus exists indicating which birth weight chart most accurately identifies growth restriction in the newborn [15, 16].

The dichotomous SGA approach, which defines a newborn as appropriate for gestational age (birth weight between the 10^th^ and 90^th^ percentile) or as SGA has inherent flaws. First, FGR is a functional placental problem, only partly related to small size. However, since measures of placental function are not often available in daily practice and consequently not feasible in large studies SGA is an important parameter used to detect FGR, albeit an imperfect one. Therefore, it is important to identify which growth chart is most accurate in predicting severe adverse perinatal outcomes [17–19]. Consequently, postnatal confirmation of growth restriction in the newborn should consider other antenatal and postnatal markers [20, 21]. Second, FGR is associated with severe adverse perinatal outcomes but there is no cut-off that adequately predicts them nor a cut-off that is without severe adverse perinatal outcomes [22–24]. Most newborns with a birth weight below the 10^th^ percentile are constitutionally small but healthy, while a significant proportion of fetuses that have experienced placental insufficiency have a birth weight above the 10^th^ percentile [25]. Not surprisingly, there is no consensus on which birth weight chart is most suited for indicating the risk of severe adverse perinatal outcomes [16, 26].

Although birth weight charts and reference curves have been extensively analyzed and compared, less attention has been given to the threshold used to define FGR and prospective large-scale studies in a low-risk population with data on severe adverse perinatal outcomes on the entire spectrum of birth weight percentiles are scarce [26–32].

We investigated the association of birth weight with severe adverse perinatal outcomes in a large-scale low-risk prospective cohort derived from the Dutch IRIS trail [33, 34]. We used birth weight percentiles as a semi-continuous measure and compared the prognostic performance for identifying newborns at risk for severe adverse perinatal outcomes using three different birth weight charts [22–24]. As the population is Dutch, we compare the Visser curve, a descriptive chart developed using the Dutch population, the Hoftiezer birth weight chart, a Dutch prescriptive birth weight chart, and the INTERGROWTH birth weight chart, a birth weight chart that was developed for international use for all populations.

## Materials and methods

### Aim of the study

To identify the newborn at risk for severe adverse perinatal outcomes across birth weight centiles in two Dutch and one international birth weight chart.

### Study design and setting

This is a secondary data-analysis of the IRIS (IUGR Risk Selection) study [33, 34]. The IRIS study is a nationwide trial examining the (cost-) effectiveness of routine third trimester ultrasonography (as compared to clinically indicated ultrasonography) in reducing severe adverse perinatal outcomes through subsequent protocolized management. The design of the IRIS study has previously been described in more detail [33]. In short, assessments in pregnant women and data-collection consisted of fetal ultrasounds, questionnaires, retrieval of information from medical records and linkage of perinatal registry data. Children were born between March 2015 and August 2016.

In the Netherlands, hospitals provide secondary and tertiary antenatal care whereas primary care midwives are independent medical practitioners who are qualified to provide full maternity care for women with low-risk pregnancies. Women receiving care from 60 participating midwifery practices and a normal 20 weeks anomaly scan and who, at that point, had a low-risk singleton pregnancy were eligible for inclusion in the IRIS study [33]. Medical conditions that require secondary or tertiary antenatal care were exclusion criteria. Medical conditions that require secondary or tertiary antenatal care were exclusion criteria. Eligible women either gave birth in the hospital if they wished to do so or in case of (risk of) complications or at home. The women who participated in the trial received either the intervention strategy (i.e., routine ultrasonography at 28–30 and at 34–36 weeks’ gestation in addition to clinically indicated ultrasonography) or the control strategy (third trimester ultrasonography only if clinically indicated). In both strategies, serial fundal height measurements were performed and detection and clinical management of suspected FGR was conducted according to a pre-defined standardized protocol [35].

At 22.8 weeks’ gestation (SD = 2.4) on average, 13,520 low risk pregnant women were included in the IRIS study between February 2015 and February 2016. For 13,001 (96.1%) infants of these 13,520 eligible women, data on birth weight and/or gestational age were available that could also be linked to data of the Netherlands’ Perinatal Registry [36]. In 48 of these infants no information on severe adverse perinatal outcomes was available. This left 12,953 newborns for our main analyses.

### Baseline characteristics and demographic variables

At inclusion midwives collected and reported pregnant women’s information on age and ethnic background, parity, maternal length, and height. Information on birth weight, gestational age, and infant sex were retrieved from the Netherlands Perinatal Registry (Perined) and/or hospital records in a subsample of 2,339 participants [33].

### Birth weight reference and charts

The Visser birth weight reference curve was used in the IRIS study as this was common practice in the Netherlands at the time [37]. This is a descriptive chart that was developed using data from the Netherlands Perinatal Registry (Perined), covering about 95% of the births in the Netherlands [36]. A one year nationwide birth cohort was used including approximately 180,000 children born in 2001 [37]. The population used had a mixed risk of adverse outcomes as the only exclusion criteria for the cohort were multiple pregnancies, antepartum stillbirths and a Hindustani ethnicity due to lower birth weights of Hindustani babies.

The Hoftiezer chart is a prescriptive chart that was developed using the data from the Netherlands Perinatal Registry (Perined) of more than 1.5 million children born in the Netherlands between 2000 and 2014. Live-born singleton infants, born to healthy mothers after uncomplicated pregnancies and a spontaneous onset of labor were included in the cohort. Exclusion criteria for the Hoftiezer chart were risk factors for FGR including maternal conditions like pre-existent hypertension and respiratory disorders and obstetric complications such as gestational hypertension and pre-eclampsia. Newborns that were born before 23 week’s gestation or after 42 weeks’ gestation were also excluded [38]. All ethnicities were included. For the Hoftiezer chart birth weight percentiles were calculated using gestational age at birth, infant sex, and birth weight in grams. This chart was implemented in the Netherlands to replace the Visser percentiles after the IRIS study was conducted.

The INTERGROWTH chart is also a prescriptive chart. It was developed to establish international charts for birth weight to enable global use. Pregnant women from eight different countries with a low risk for FGR were selected for the cohort [39]. 20,486 children were included from whom sex- and gestational age specific INTERGROWTH birth weight charts were derived [40, 41].

### Main outcome

The main outcome of the IRIS study were severe adverse perinatal outcomes. These outcomes were selected using previous research and expert opinion [33]. Information on severe adverse perinatal outcomes was based on data derived from Perined and from hospital records by 5 trained research assistants. Double data entry analyses based on a subsample of 111 women revealed an adequate data entry quality as reported earlier [33, 34]. The dichotomous main outcome was a composite measure of severe adverse perinatal outcomes that are associated with FGR. The main outcome was defined as one or more of the following severe adverse outcomes occurring from the moment of inclusion up to seven days after birth:

1. Perinatal death occurring from 28 weeks’ gestation until one week after birth
2. Apgar score < 4 at 5 minutes
3. Impaired consciousness (coma, stupor, or decreased response to pain)
4. Asphyxia, defined as cord blood arterial base excess of less than minus 12 mmol/L
5. Seizures on two or more occasions within 72 h after birth
6. Assisted ventilation for more than 24 h via endotracheal tube initiated within 72 h of birth
7. Septicemia ascertained by blood culture
8. Meningitis ascertained by cerebrospinal fluid culture
9. Bronchopulmonary dysplasia (BPD), requiring oxygen at a postnatal gestational age from 36 completed weeks and ascertained by radiography
10. Intraventricular hemorrhage grade three or four and diagnosed by ultrasound or autopsy
11. Cystic periventricular leukomalacia (PVL) ascertained by ultrasonography
12. Necrotizing enterocolitis diagnosed by radiography, surgery, or autopsy

### Statistical analyses

For each birth weight chart gestational age specific standard deviation scores (SDSs) were calculated using formulas of the Visser, Hoftiezer and INTERGROWTH charts. For each chart, the derived birth weight centiles were divided into the following centile groups: < p3, p3- < p5, p5- < p10, p10- < p25, p25- < p75, p75- < p90, p90- < p95, p95- < p97 and > p97. The distribution and rates of severe adverse perinatal outcomes in the birth weight centile groups were examined to investigate the diagnostic performance of each birth weight chart per centile group. Sensitivity and specificity rates and likelihood ratios were calculated for the various birth weight centile cut-offs. To explore the degree of accuracy by which the birth weight charts predict severe adverse perinatal outcomes, ROC curves were calculated. The area under the curve (AUC) of a ROC curve illustrates the diagnostic ability of the birth weight chart. If the AUC is 0.5 this means that the birth weight chart does not discriminate between low and high risk of severe adverse perinatal outcomes. An AUC between 0.7 and 0.8 is considered an acceptable diagnostic performance and an AUC above 0.8 is considered a good diagnostic performance.

Analyses were carried out in Statistical Package for the Social Sciences (SPSS) 26. The Hoftiezer SDSs were calculated in R, version 3.02. The birth weight percentiles from the INTERGROWTH chart were calculated using the “Neonatal Size Calculator for newborn infants between 24^+0^- and 42 + 6-weeks’ gestation” (https://intergrowth21.tghn.org/newborn-size-birth/).

## Results

Of the 12,953 women, 215 (1.7%) gave birth to a newborn that experienced at least one severe adverse perinatal outcome. The most common adverse outcome was asphyxia which occurred 144 times (1.11%). Table 1 presents participants’ baseline characteristics. The relative frequencies of the elements of the composite “severe adverse perinatal outcomes” are shown in Table 2.

**Table 1:** Baseline characteristics (*n* = 12,953)

*Maternal characteristics*	*N*	M (SD)/%^a^
Maternal age, years	12,953	31.0 (4.4)
Pre-pregnancy BMI		
Underweight (< 18.5)	415	3.2
Normal	8548	66.0
Overweight/obese (> 25)	3827	29.9
Missing	163	1.3
Ethnicity		
Dutch	9,721	75.0
Other western	1,331	10.3
Non-western	1,893	14.6
Missing	8	0.1
Smoking during pregnancy		
No	11,101	85.7
Yes	1821	14.1
Missing	31	0.2
Educational level		
Low	1279	9.9
Medium	4531	35.0
High	6912	53.4
Missing	231	1.8
Parity		
Nulliparous	6243	48.2
Multiparous	6596	50.0
Missing	114	0.9
***Infant characteristics***		
Birth weight, grams	12,953	3485 (510.9)
Gestational age at birth, weeks^b^	12,950	39.7 (1.6)
Infant sex		
Boys	6589	50.9
Girls	6363	49.1
Missing	1	0.0

*BMI* body mass index. Due to rounding percentages do not always exactly add up to 100.0%

^a^Numbers differ due to missing values

^b^Gestational age at birth had missing values (*n* = 3, 0.0%)

**Table 2:** Severe adverse perinatal (composite) outcomes^a^

Severe adverse perinatal outcome	*N (%)*
Perinatal death	24 (0.19)
Apgar score < 4 at 5 min	41 (0.32)
Impaired consciousness	16 (0.12)
Asphyxia	144 (1.11)
Seizures on two or more occasions	12 (0.09)
Assisted ventilation for more than 24 h	44 (0.34)
Septicemia	11 (0.08)
Meningitis	3 (0.02)
Bronchopulmonary dysplasia	1 (0.01)
Intraventricular hemorrhage grade three or four	1 (0.01)
Cystic periventricular leukomalacia	0 (0.00)
Necrotizing enterocolitis	2 (0.02)
Composite of severe adverse perinatal outcomes	215 (1.66)

^a^The primary dichotomous clinical outcome, i.e., severe adverse perinatal outcome, was a composite measure comprising one or more of the 12 severe adverse perinatal outcomes up to 7 days after birth

**Fig. 1:**
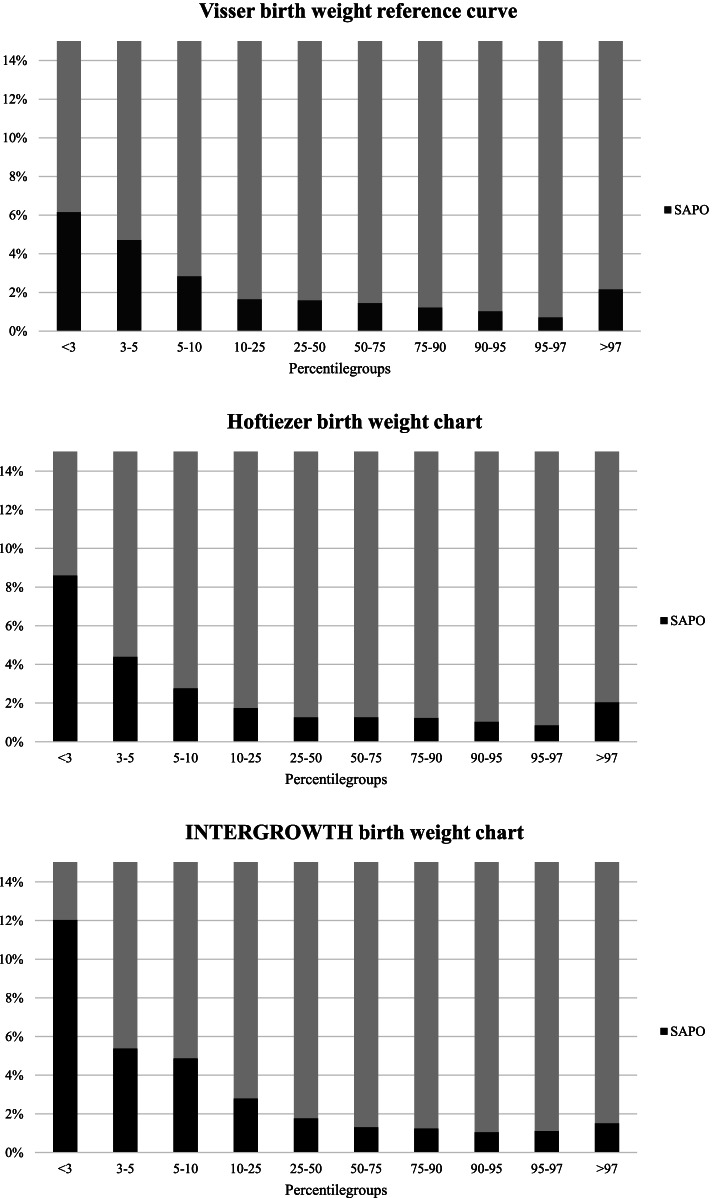
Severe adverse perinatal outcomes throughout the birth weight percentile groups

In Fig. 1, the distribution of severe adverse perinatal outcomes throughout the birth weight percentile groups is shown. Visual inspection of the distributions of severe adverse perinatal outcomes by birth weight centile groups indicates a curvilinear pattern for all birth weight charts, with the highest rates of severe adverse perinatal outcomes occurring among SGA newborns (birth weight below the 10^th^ percentile). Newborns with a birth weight below the 3^rd^ percentile had particularly high rates of severe adverse perinatal outcomes.

Figure 1 and Table 3 also show that among the SGA newborns rates of severe adverse perinatal outcomes decreased with increasing birth weight centiles. The figure also illustrates that large for gestational age (LGA) newborns above the 97^th^ percentile had a slightly increased rate of severe adverse perinatal outcomes for the Visser and Hoftiezer charts but to a lesser degree than SGA newborns.

**Table 3:** Rates of severe adverse perinatal outcome, sensitivity, specificity, positive and negative likelihood ratios per centile group of the Visser, INTERGROWTH, and Hoftiezer birth weight standard

*Centile group*	***Visser***		***INTER-*** ***GROWTH***			***Hoftiezer***	
	*N*	*SAPO*^*a*^*, n (%)*		*N*	*SAPO*^*a*^*, n (%)*	*N*	*SAPO*^*a*^*, n (%)*
< p3	211	13 (6.2)	108		13 (12.0)	349	30 (8.6)
p3 to < p5	191	9 (4.7)	93		5 (5.4)	250	11 (4.4)
p5 to < p10	599	17 (2.8)	267		13 (4.9)	579	16 (2.8)
p10 to < p25	1823	30 (1.6)	1002		28 (2.8)	1886	33 (1.7)
p25 to < p75	6637	101 (1.5)	5877		88 (1.5)	6477	82 (1.3)
p75 to < p90	1973	24 (1.2)	2821		35 (1.2)	2014	25 (1.2)
p90 to < p95	681	7 (1.0)	1243		13 (1.0)	677	7 (1.0)
p95 to p97	403	5 (1.2)	545		5 (0.9)	272	3 (1.1)
> p97	435	9 (2.1)	993		15 (1.5)	393	8 (2.0)
*Sensitivity, % (95%CI) *^*a*^							
< p3	6.0 (3.3; 10.1)			6.1 (3.3; 10.1)		14.0 (9.6; 19.3)	
< p5	10.2 (6.5; 15.1)			8.4 (5.0; 12.9)		19.1 (14.1; 25.0)	
< p10	18.1 (13.2; 24.0)			14.4 (10.0; 19.8)		26.5 (20.7; 32.9)	
< p25	32.1 (25.9; 38.8)			27.4 (21.6; 33.9)		41.9 (35.2; 48.8)	
> p75	20.9 (15.7; 27.0)			31.6 (25.5; 38.3)		20.0 (14.9; 26.0)	
> p90	9.8 (6.2; 14.5)			15.3 (10.8; 20.9)		8.4 (5.0; 12.9)	
> p95	6.5 (3.6; 10.7)			9.3 (5.8; 14.0)		5.1 (2.6; 9.0)	
> p97	4.2 (1.9; 7.8)			7.0 (4.0; 11.3)		3.7 (1.6; 7,2)	
*Specificity, % (95% CI)*^*a*^							
< p3	98.4 (98.2; 98.7)			99.3 (99.1; 99.4)		97.5 (97.2; 97.8)	
< p5	97.0 (96.7; 97.3)			98.6 (98.3; 98.8)		95.6 (95.2; 96.0)	
< p10	92.5 (92.0; 92.9)			96.6 (96.2; 96.9)		91.2 (90.7; 91.7)	
< p25	78.4 (77.7; 79.1)			88.9 (88.4; 89.5)		76.6 (75.8; 77.3)	
> p75	72.9 (72.2; 73.7)			56.6 (55.7; 57.4)		73.9 (73.1; 74.6)	
> p90	88.2 (87.7; 88.8)			78.4 (77.7; 79.1)		89.6 (89.0; 90.1)	
> p95	93.5 (93.1; 94.0)			88.1 (87.5; 88.7)		94.8 (94.4; 95.2)	
> p97	96.7 (96.3; 97.0)			92.3 (91.8; 92.8)		97.0 (96.7; 97.3)	
*Positive likelihood ratio (95%CI)*^*a*^							
< p3	3.89 (2.26; 6.71)			8.10 (4.61; 14.24)		5.55 (3.91; 7.87)	
< p5	3.43 (2.28; 5.16)			5.83 (3.66; 9.27)		4.33 (3.25; 5.78)	
< p10	2.40 (1.80; 3.21)			4.20 (3.00; 5.89)		3.00 (2.38; 3.77)	
< p25	1.48 (1.22; 1.81)			2.48 (1.98; 3.09)		1.79 (1.52; 2.10)	
> p75	0.77 (0.60; 1.00)			0.73 (0.60; 0.89)		0.77 (0.59; 1.00)	
> p90	0.83 (0.55; 1.25)			0.71 (0.52; 0.98)		0.80 (0.51; 1.25)	
> p95	1.01 (0.60; 1.68)			0.78 (0.51; 1.19)		0.99 (0.56; 1.77)	
> p97	1.25 (0.66; 2.39)			0.91 (0.56; 1.49)		1.23 (0.62; 2.44)	
*Negative likelihood ratio (95% CI)*^*a*^							
< p3	0.95 (0.92; 0.99)			0.95 (0.92; 0.98)		0.88 (0.84; 0.93)	
< p5	0.93 (0.88; 0.97)			0.93 (0.89; 0.97)		0.85 (0.79; 0.90)	
< p10	0.89 (0.83; 0.94)			0.89 (0.84; 0.94)		0.81 (0.74; 0.87)	
< p25	0.87 (0.79; 0.95)			0.82 (0.75; 0.89)		0.76 (0.68; 0.85)	
> p75	1.08 (1.01; 1.16)			1.21 (1.10; 1.33)		1.08 (1.01; 1.16)	
> p90	1.02 (0.98; 1.07)			1.08 (1.02; 1.14)		1.02 (0.98; 1.07)	
> p95	1.00 (0.96; 1.04)			1.03 (0.99; 1.07)		1.00 (0.97; 1.03)	
> p97	0.99 (0.96; 1.02)			1.01 (0.97; 1.05)		0.99 (0.97; 1.02)	

Numbers indicate absolute numbers (n) and percentages (%)

^a^Severe adverse perinatal outcomes

The distributions of severe adverse perinatal outcomes in the centile groups of each birth weight chart and the sensitivity and specificity rates for each cut-off are shown in Table 3. The sensitivity rates were low for all three birth weight charts, especially in the < p3 percentile group (6.0 – 14.0%). The specificity rates were higher, especially in the < p3 percentile group (97.5 – 99.3%). The Hoftiezer birth weight chart had the highest sensitivity rates in all three percentile groups below p10 (< p3, p3- < p5 and p5- < p10). When comparing the positive likelihood ratios (Table 3), the INTERGROWTH birth weight chart seems the most accurate for predicting severe adverse perinatal outcomes. For the newborns in the < p3 birth weight percentile group the positive likelihood ratio for severe adverse perinatal outcomes was higher when the INTERGROWTH birth weigh chart was used (8.10 versus 3.89 for the Visser reference curve and 5.55 for the Hoftiezer birth weight chart). For none of the birth weight charts and in none of the birth weight percentile groups the positive likelihood ratio for severe adverse perinatal outcomes was higher than 10, showing that the diagnostic accuracy of all three birth weight charts was low or moderate at the most. This pattern of results was also shown by the calculated areas under the curve (AUC), indicating the degree of accuracy with which birth weight percentiles detect severe adverse perinatal outcomes with all AUC’s below 0.7 (Visser 0.57 (0.53; 0.62), INTERGROWTH 0.61 (0.57; 0.65) and Hoftiezer 0.61 (0.57; 0.65)). The negative likelihood ratios were high, showing that birth weight > p3 as a cut-off is not a good method to identify newborns with a low risk of severe adverse perinatal outcomes.

## Discussion

### Main findings

For each of the three charts used, high rates of severe adverse perinatal outcomes were observed at the extremes of the distribution of the birth weight centiles. However, the discriminative ability was poor when the cut-off at the 10^th^ percentile (SGA) was used. Similar curvilinear patterns of the link between birth weight and severe adverse perinatal outcomes have been reported for perinatal mortality curves that were presented by Vasak et al [42]. With decreasing weight percentile at birth, the risk of severe adverse perinatal outcomes increased. Newborns with a birth weight above the 97^th^ percentile also had slightly higher rates of severe adverse perinatal outcomes.

The incidence of severe adverse perinatal outcomes in the < p3 percentile group was highest. This is in line with the ideas of the international consensus definitions for FGR and growth restriction in the newborn (GRN) [21]. The severe adverse perinatal outcomes incidence < p3 percentile groups was highest when the INTERGROWTH birth weight chart was used (12.0% versus 6.2% for Visser and 8.6% for Hoftiezer). Part of the explanation for this may be that the INTERGROWTH chart considers lower absolute birth weights indicative of SGA, as compared to the two Dutch birth weight charts [37–39, 41]. When the INTERGROWTH chart was developed only small differences were seen in birth weight between the eight populations. These differences were not considered meaningful and therefore one generalized INTERGROWTH chart was calculated for international use [41]. However, the Dutch population is characterized by relatively high birth weights [38, 43, 44]. It is likely that this has influenced the higher rate of severe adverse perinatal outcomes among SGA newborns as identified by the INTERGROWTH chart. It is likely that the newborns that are identified as (severe) SGA using the INTERGROWTH chart have a higher risk of severe adverse perinatal outcomes. On the other hand, there will also be more newborns that have experienced suboptimal conditions in utero and that are growth restricted but that will remain unidentified because their birth weight is not below the 10^th^ percentile.

For all three charts the area under the curve was smaller than 0.7. The Hoftiezer birth weight chart had somewhat higher sensitivity rates for the < p3, p3- < p5 and p5- < p10 percentile groups but lower specificity rates. Rates of sensitivity and specificity vary with the prevalence of a certain disease [45]. The low sensitivity rates observed may be partly due to the low-risk status of the IRIS study population. The incidence of severe adverse perinatal outcomes was only 1.7% which may have attributed to the low sensitivity rates. This is why likelihood ratio’s were also calculated, which are less dependent on the incidence of severe adverse perinatal outcomes [46]. In sum, the INTERGROWTH and the Hoftiezer predictive birth weight charts performed slightly better than the descriptive Visser birth weight reference curve but the overall diagnostic accuracy in identifying the risk of severe adverse perinatal outcomes of all three birth weight charts in this low risk population was low.

### Interpretation

Our study is one of the few studies that analyzed the occurrence of severe adverse perinatal outcomes across the full range of birth weight percentiles and that suggests that birth weight is curvilinearly related to severe adverse perinatal outcomes [1, 22, 47–49].

There is no specific birth weight percentile that reflects a safe weight for gestational age with regard to placental function and severe adverse perinatal outcomes, although we observed a gradient in which newborns with the lowest birth weight have the highest risk of severe adverse perinatal outcomes. This is in line with the idea that p10 should not be the cut-off for healthy size [17]. The same pattern was observed above the p90, but to a lesser degree. These findings are in line with previous research [15, 50]. When using the SGA/LGA cut-off, many newborns will be classified to be of abnormal size without being at risk for severe adverse perinatal outcomes. There is also a group of newborns that is not classified as abnormal size but that did experience suboptimal conditions in utero. These newborns might not receive adequate monitoring and care after birth. This confirms that more efforts are needed to improve the prediction of newborns at risk of adverse outcomes before and after birth. Functional antenatal parameters for placental function, if available, may be added to the biometric measurement [17, 51].

Based on a Delphi consensus study that was performed among neonatologists to determine a definition of growth restriction in the newborn it was concluded that a birth weight < p3, on any birth weight chart, should be used to determine the risk of severe adverse perinatal outcomes. However, next to birth weight < p3 other factors should also be considered [21]. Candidates include other biometric data, body composition, metabolic data and biomarkers that yet need to be determined. However, it should also be borne in mind that high specificity is important to avoid overdiagnosis in healthy newborns. A similar Delphi consensus definition for FGR was published in 2016 [17]. The definition comprised not only fetal biometric (size) measurements but also measurements of placental function. According to this definition, FGR can be diagnosed based on functional markers of the placenta such as ultrasound Doppler measurements (reflecting vascular resistance in the umbilical cord and middle cerebral artery) combined with estimated fetal weight or abdominal circumference crossing centiles, independent of fetal size [17].

Besides biometric and doppler measurements there are also other markers that can be used to detect impaired fetal condition or placental insufficiency such as mother-reported reduced fetal movements, electronic fetal monitoring, the amount of amniotic fluid, metabolites in maternal serum and placental biomarkers [18, 52]. However, all these markers have similar shortcomings and are not suitable for the prediction of the risk of severe adverse perinatal outcomes if used as stand-alone. Perhaps a prediction model for severe adverse perinatal outcomes should be made that combines several of these markers.

Interestingly, in previous trials including the IRIS study, routine third trimester ultrasonography generally increased the detection of SGA, but it did not reduce the rate of severe adverse perinatal outcomes compared to clinically indicated ultrasonography [25, 34, 53]. This underlines that both fetal biometry and birth weight are imperfect proxies for the risk of severe adverse perinatal outcomes and that other factors should always be taken into account.

Some suggest developing birth weight charts that better reflect growth potential, the so called ‘customized growth references’, for example by adjusting for maternal physiological characteristics like maternal height [54–57]. In the IRIS study slow fetal growth, defined as a decline in abdominal circumference of at least 20 percentiles was used as a proxy for FGR. It would be interesting to relate slow fetal growth, for example from 20 weeks onwards, to the risk of severe adverse perinatal outcomes [58].

### Strengths and limitations

A strength of this study is its rich dataset that is based on questionnaire data, perinatal registry data, and hospital records data all collected within in a prospective large-scale nationwide study.

Our study also has some limitations. The population used for analyses is a low-risk Dutch population that received primary antenatal care at the time of inclusion. Two of the three analyzed birth weight charts were also Dutch charts. In this low-risk sample only 1.7% of the neonates had severe adverse perinatal outcomes. It is conceivable that rates of severe adverse perinatal outcomes are higher in the general population. Therefore, the test performance (e.g., sensitivity rates) of the birth weight charts might have been underestimated based on the current sample, as sensitivity rates vary with the prevalence of a certain disease [45]. However, it is rather unlikely that this influenced the observed differences between the birth weight charts in their ability to identify severe adverse outcomes. Given the findings of this study that there is no single cut-off that discriminates between groups of high and low risk, we consider that our findings are generalizable to other contexts, nonetheless.

In addition, only severe, and immediate adverse perinatal outcomes occurring during the first seven days after birth were included in the analyses. There is an increasing body of evidence about the subtle long term consequences for the growth restricted babies [10, 11]. These long term consequences are not included in our analyses.

Finally, interventions were done if FGR was suspected. These interventions will probably have had an influence on the outcomes of the growth restricted newborns.

## Conclusions

While we observed a gradient in which newborns with the lowest birth weight, on either chart, have the highest risk of severe adverse perinatal outcomes, no single cut-off discriminated clearly between newborns at low- or high-risk. All examined birth weight charts demonstrated a poor clinical utility as a single predictor in identifying (the risk of) severe adverse perinatal outcomes. More research should be performed to find sensitive markers identifying suboptimal conditions in utero and impaired fetal growth and development, also applicable when the growth restriction is suspected as late as after birth. Currently, techniques for Doppler ultrasound (for antenatal diagnosis) and biomarkers measurements (for antenatal and postnatal diagnosis) are being studied and show promising results in predicting and reducing severe adverse perinatal outcomes [17, 18, 52]. In addition, more efforts are needed to construct better birth weight charts that reflect the growth potential, for instance by adjusting for maternal physiological characteristics. A prediction model that includes multiple markers for fetal growth and placental function could also contribute to the prediction and management of fetal growth restriction.

## Data Availability

The data presented in this study are available on request from the corresponding author. The data are not publicly available due to the ethical approval provided for the IRIS study, which does not allow publication of individual participant level data.

## Abbreviations

FGR: Fetal growth restriction
SGA: Small for gestational age
LGA: Large for gestational age
SAPO: Severe adverse perinatal outcomes
Perined: Netherlands perinatal registry
BPD: Bronchopulmonary dysplasia
PVL: Cystic periventricular leukomalacia
SDSs: Standard deviation scores
AUC: Area under the curve
ROC curve: Receiver operating characteristic
